# Adaptive LINE-P: An Adaptive Linear Energy Prediction Model for Wireless Sensor Network Nodes

**DOI:** 10.3390/s18041105

**Published:** 2018-04-05

**Authors:** Faisal Ahmed, Gert Tamberg, Yannick Le Moullec, Paul Annus

**Affiliations:** 1 Thomas Johann Seebeck Department of Electronics, Tallinn University of Technology, Tallinn 12616, Estonia; paul.annus@ttu.ee; 2 Department of Cybernetics, Tallinn University of Technology, Tallinn 12616, Estonia; gert.tamberg@ttu.ee

**Keywords:** WSN, energy harvesting, energy prediction

## Abstract

In the context of wireless sensor networks, energy prediction models are increasingly useful tools that can facilitate the power management of the wireless sensor network (WSN) nodes. However, most of the existing models suffer from the so-called fixed weighting parameter, which limits their applicability when it comes to, e.g., solar energy harvesters with varying characteristics. Thus, in this article we propose the Adaptive LINE-P (all cases) model that calculates adaptive weighting parameters based on the stored energy profiles. Furthermore, we also present a profile compression method to reduce the memory requirements. To determine the performance of our proposed model, we have used real data for the solar and wind energy profiles. The simulation results show that our model achieves 90–94% accuracy and that the compressed method reduces memory overheads by 50% as compared to state-of-the-art models.

## 1. Introduction

### 1.1. Energy Harvesting and Prediction Models

Energy harvesting (EH) is a promising technology that became a hot topic in the scientific community during the last few decades; however, EH is still a least explored area, especially at the micro and nano power levels. In particular, EH at the micro level is quite useful to power ultra-low-power sensor nodes. EH introduces various paths of research for prolonging the lifespan of wireless sensor network (WSN) nodes, either as an energy harvester with buffered energy storage (e.g., battery or super-capacitor) or directly (e.g., without energy storage) for autonomous devices. Several EH approaches, presented in the literature, exploit solar, wind, or thermal energy. 

EH is a good alternative solution for those applications that are implemented once and become operational for longer periods; examples include environmental monitoring, structural monitoring, etc. Furthermore, WSN applications can benefit from EH to extend the life of the node or network [1]. Generally, numerous methods associated with EH have been discussed in the literature. These include energy-aware protocols, duty cycle management, task scheduling, transient computing (TC) in stand-alone mode (which performs transmission when the energy is available with or without any power source battery), data prediction [2], as well as mobility, which can reduce the power consumption if mobility incurs low overheads [3].

According to the literature, EP plays an important role for EH in the context of WSNs. Energy prediction for non-controllable energy source seeks to provide information about the upcoming available energy based on past records (profiles) and/or current values. EP mechanisms increase the system’s efficiency [4] because they enable more careful utilization of the available energy as well as the dynamic execution of tasks depending upon the estimation of the energy available in the next time slots. Given the importance of EP, it is necessary to propose accurate sets of energy prediction models in order to increase the performance and other important processes for better quality of service.

In this article, we first briefly recall the limitations of most existing EP models due to the fixed-weighting parameter issue; thereafter, we suggest a solution with an adaptive weighting factor based on the energy profiles. In [5], we discussed how most of the energy prediction models such as EWMA, WCMA, ASEA, PRO-Energy, QL-SEP, and LINE-P (all cases) are dependent on a fixed weighting parameter; however, these solutions are not always suitable for real implementations with many various types (and hence characteristics) of e.g., solar energy harvesters. 

In addition, the proposed Adaptive LINE-P (all cases) model estimates the energy over three different time periods, namely shorter, medium, and longer, and uses variable-length timeslots. The proposed prediction model improves the prediction accuracy and minimizes the error between the harvested energy and stored profiles. Furthermore, in this article we propose a compression method that reduces the size of the stored energy profiles by 50% in order to reduce memory overheads. 

### 1.2. Contribution

Our contribution can be summarized as follows:

- We propose and evaluate enhancements to the existing LINE-P (all cases) model; we name the resulting new model “Adaptive LINE-P (all cases)”; namely,
  ○ we propose an adaptive parameter to address the fixed weighting parameter issue that is found in most existing energy prediction models, specifically when targeting solar energy harvesters;
  ○ we propose a profile compression technique that can be integrated in any energy prediction model.
- Our results show that the proposed enhancements achieve up to 98% accuracy (non-compressed profile) and up to 90% accuracy but with a 50% reduction of the memory requirements when using the compressed profile method, as compared to the state of the art.

The rest of the article is formulated as follows. Related work is presented in Section 2. The proposed Adaptive LINE-P (all cases) is detailed in Section 3. The comparative performance evaluation of the models is discussed in Section 4. Finally, we briefly conclude in Section 5.

## 2. Materials and Methods 

Here, we discuss the state of the art regarding the fixed kernel parameter issue, variable length time slots, and dynamic or adaptive energy prediction models related to the domain of WSNs. 

### 2.1. Non-Adaptive Energy Prediction Models

In [5] the authors presented three cases of LINE-P (linear energy prediction model) that are based on the sampling and approximation theory. The authors showed that LINE-P (all cases) is more accurate, has a lower complexity, and is energy-efficient in terms of computation as compared to other non-adaptive EP models.

However, the above comparison did not include the latest extensions, namely Pro Energy VLT and IPro-Energy. Thus, in this sub-section we first briefly introduce LINE-P and then discuss Pro Energy VLT and IPro Energy. 

#### 2.1.1. LINE-P

We first briefly introduce LINE-P. The detailed mathematical derivations can be found in [5].

##### Linear Energy Prediction LINE-P (Case I)

The main aim when designing LINE-P was to minimize the computational complexity while maintaining similar accuracy to other models. 

If we have the samples $f_{l}\left(l=1,\ldots ,k\right)$ from k previous days, we utilize this information as the basis for EP. Here, vector b defines a symmetric kernel and the parameter vector a, where $a_{k}=0$ for k≤0, generates a one-sided kernel with the correspondent sampling operator:

(1) $$(S_{PREDI;b}f)\left(j\right):=\sum_{k=1}^{m}b_{k}f\left(j-k\right)+\sum_{k=-m}^{0}b_{k}\;f_{l}\left(j-k\right)+CDIF_{PREDI;a;b;l}\left(j\right),$$

where the correction term $CDIF_{PREDI;b}$ in Equation (1) is given as

(2) $$CDIF_{PREDI;a;b;l}\left(j\right):=CT_{PREDI;a;b}\left(\sum_{k=1}^{n}a_{k}f\left(k-i\right)-\sum_{k=1}^{n}a_{k}\;f_{l}\left(j-k\right)\right),$$

with the multiplier $CT_{PREDI;b}$ defined as:

(3) $$CT_{PREDI;a;b}:=\sum_{k=-m}^{0}b_{k}.$$

Equation (1) is used to estimate the energy based on the next time slot, specifically for LINE-P (Case I), and Equations (2) and (3) are the substitution factors of Equation (1).

##### Linear Energy Prediction LINE-P (Case II)

In this case, we proposed a model that performs energy estimation with only *n* previous samples from the same day. This case is dependent on only one variable, i.e., *a*:

(4) $$(S_{PREDII;a}f)\left(j\right):=\overset{m}{\underset{k=1}{\sum }}a_{k}f\left(j-k\right).$$

##### Linear Energy Prediction LINE-P (Case III)

The third case is similar to Case I; the only difference is in $CT_{PREDIII;b}$ as shown in Equation (7).

(5) $$(S_{PREDIII;b}f)\left(j\right):=\sum_{k=1}^{m}b_{k}f\left(j-k\right)+\sum_{k=-m}^{0}b_{k}\;f_{l}\left(j-k\right)+CDIF_{PREDIII;b;l}\left(j\right),$$

where the correction term $CDIF_{PREDIII;b:l}$ is in Equation (6),

(6) $$CDIF_{PREDIII;b;l}\left(j\right):=CT_{PREDIII;b\;}\left(\sum_{k=1}^{m}b_{k}f\left(j-k\right)-\sum_{k=1}^{m}b_{k}\;f_{l}\left(j-k\right)\right),$$

with the multiplier $CT_{PREDIII;b}$

(7) $$CT_{PREDIII;b}:=\frac{\sum_{k=-m}^{0}b_{k}\;}{\sum_{k=1}^{m}b_{k}\;}.$$

We select, from the *k* previous days, day *l* for which the absolute value of the correction term $CDIF_{PREDIII;b;l}$ is minimal and consider the values $f_{l}$ from that day.

#### 2.1.2. Pro-Energy-VLT

In this subsection, Pro-Energy-VLT is discussed. In [6], the authors presented Pro-Energy with variable-length timeslots (Pro-Energy-VLT), based on the Pro-Energy model. In particular, the author proposed a perceptually important point (PIP) technique to calculate the variable size timeslots such as 30, 60, and 90 min [6], as compared to their original design, which was fixed to 30-min data intervals [6]. The authors revealed that Pro-energy-VLT increases the prediction accuracy while reducing the memory and the energy overhead of energy forecasting [6]. However, the authors used two fixed weighting factors α and γ in their algorithms to estimate energy for the next time slot over short and medium data intervals. As mentioned earlier, such a fixed tuning parameter is not compatible with various solar energy harvesters with different characteristics. 

#### 2.1.3. IPro-Energy

**Figure 1:**
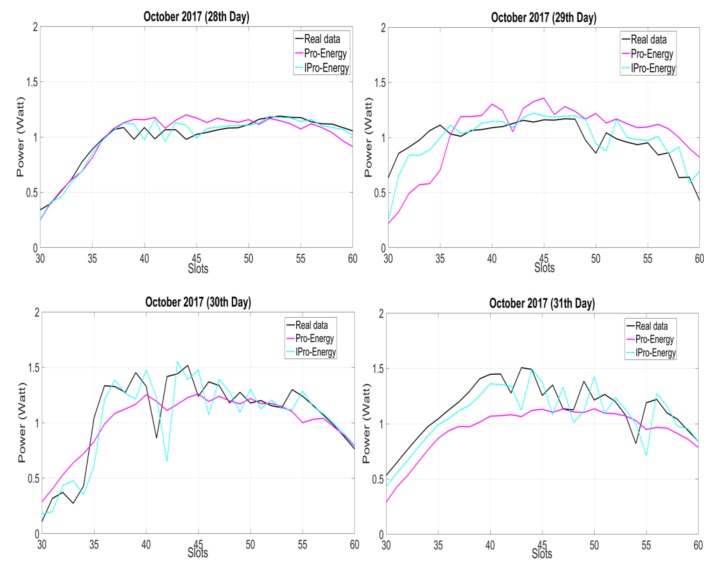
Illustration of a four-day comparative analysis of IPro-Energy with Pro-Energy.

We now discuss IPro-Energy, which is also based on the Pro-Energy model. In [7], the authors of IPro-energy highlighted its two main features. Firstly, IPro-Energy uses a weighted profile (WP) technique to compensate for inconsistency in the weather behavior. Secondly, the authors showed that the model has a low complexity in terms of execution time, and low requirements in terms of storage data. We have conducted a simulation test of IPro-energy and compared its results with Pro-Energy; we found that, indeed, IPro-Energy yields better results than Pro-Energy, as shown in Figure 1. In order to quantify the prediction error, we have used two classical measures, namely MAE (mean absolute error) and MSE (mean square error), as shown in Table 1. 

Although the results yielded by IPro-Energy are better than those of Pro-Energy, the former relies on a more complex model and the execution times are much higher than those of Pro-Energy. This is because both the basic Pro-Energy model and the new features of IPro-Energy have to be executed. In particular, IPro-Energy introduces an additional weighting factor, *W_f_*, which lies at [0, 1]. Thereafter, based on *W_f_* and the *r* weighting factor (which has a 0.5 fixed value), the authors calculated the smarting factor (*S*) in Equation (8). Then, for predicting the energy based on the next timeslots, Equation (8) is inserted into Equation (9):

(8) $$S=r\;\left(\frac{\left(C_{t}-C_{t-1}\right)}{(C_{t}+C_{t-1})/2}\right)\;C_{t-1}.$$

The expected energy is denoted by *C_t_*_+*i*_ for the timeslot *t*+*i* of the current day,

(9) $$C_{t+i}=W_{f}C_{t}+\left(\left(1-W_{f}\right)\;WP_{t+i}\right)+S,$$

where *WP* is expressed as a combination of the previously observed most similar days.

Apart from the fixed parameter weighting factor-based models, there are several ANN-based algorithms available in the literature that estimate the energy based on a short-term energy prediction. In [8], the authors proposed a method for an adaptive neural network model. They used sliding window training with window sizes of three, four, and five months’ data. In particular, their simulation results show that the five-month window size was the best simulation. In addition, they show that it is good to have a larger window size for training purposes as fewer data decreases the prediction quality. However, even a window size of three months of data would be too big for the microcontroller‘s (MSP430FR5739 and MSP430G2) memory targeted in our work and in [9] it was concluded that such ANN-based models are not adaptive and not more reliable than EWMA and WCMA algorithms. 

There are others approaches to reducing the energy consumption and management in WSNs that work by estimating the energy, i.e., route selection schemes [9] and adaptive duty cycling [10]. Furthermore, in [11] the authors investigate the distributed sampling rate adaptation method in the multi-sensor implemented wireless devices to assign data capturing tasks among them based on the remaining energy network participation and correlations. In addition, they proposed effective mechanisms to utilize the ability of wireless devices to monitor a few selected points in a certain area. In [12], the authors present joint channel selection and routing schemes for multi-channel WSNs that apply duty cycling to sustain energy. The experimental tests and simulation show that the proposed schemes reduce overhearing by approximately 60% with two channels without affecting network performance. Furthermore, the researchers exploited some other techniques for minimizing energy consumption, for instance data compression and source coding [13], transmitting power control and distributed sampling rate adaptation for WSNs [14]. 

In the following, we discuss adaptive parameter weighting factor-based models for solar energy harvesting in the context of WSNs. 

### 2.2. Adaptive Energy Prediction Model

In this section we discuss UD-WCMA, the only dynamic or adaptive weighting factor-based EP model that aims at better tracking variations in the generated energy (due to, e.g., weather conditions). 

UD-WCMA [15] is developed based on the WCMA structure; it introduces a time-varying weighting parameter *G*_1_(*n* + 1)*.* This gain is adapted depending on the variations in the reference profiles stored in the memory. In addition, the energy prediction is ensured by combining the information collected from the last observations *θ*(*n*) with the mean value *µ_d_*(*n* + 1) of the harvested energy from the stored profiles.

Mathematical expression of the dynamic schemes in the UD-WCMA prediction model is as follows:

(10) $$\hat{x}\;\left(n+1\right)=G_{1}\left(n+1\right)\theta \left(n\right)+\left[1-G_{1}\left(n+1\right)\right]GAP{µ}_{d}\left(n+1\right),$$

where

(11) $$G_{1}\left(n+1\right)=\frac{\sigma \left(n+1\right)}{2\left(\sigma \left(n+1\right)+\sigma_{1}\left(n+1\right)\right)}.$$

In Equation (10) *σ* represents the standard deviation of the irradiance levels of the stored profiles at time *n* + 1 with respect to the mean value. Subsequently, *σ*_1_ is also a standard deviation that indicates the energy variation in the stored profiles between the time slots *n* and *n* + 1. They are defined, for *i* = {1, …, *d*}, by:

(12) $$\sigma \left(n+1\right)=\sqrt{\frac{1}{d}\sum_{i=1}^{d}{\left(x_{i}\left(n+1\right)-{µ}_{d}\left(n+1\right)\right)}^{2}}$$

and

(13) $$\sigma_{1}\left(n+1\right)=\sqrt{\frac{1}{d}\sum_{i=1}^{d}{\left(\Delta_{1i}\left(n+1\right)-{µ}_{d}\left(n+1\right)\right)}^{2}},$$

where

(14) $$\Delta_{1i}\left(n+1\right)=x_{i}\left(n+1\right)-x_{i}\left(n\right)$$

and

(15) $${µ}_{1}\left(n+1\right)=\frac{1}{d}\sum_{i=1}^{d}\Delta_{1i}\left(n+1\right).$$

In order to further increase the accuracy and robustness of the model, especially for dealing with inconsistent weather, the author in [15] proposed replacing the last observation *θ*(*n*) with a weighted linear combination of the last observation and the closest energy pattern in memory denoted by $x_{i}\left(n+1\right)$. Moreover, the linear combination is weighted by an adaptive factor *G*(*n* + 1) depending on the variation of the current day measurements, as follows:

(16) $$\hat{x}\left(n+1\right)=G_{1}\left(n+1\right)\left[G\left(n+1\right)\theta \left(n\right)+1-G\left(n+1\right)x_{i}\left(n+1\right)\right]+(1-G_{1}\left(n+1\right))GAP{µ}_{d}\left(n+1\right),$$

where

*G*_1_(*n* + 1) = *G*_1_(*n* + 1) + *G*_2_(*n* + 1) (17)

and

(18) $$G_{2}\left(n+1\right)=\frac{\sigma \left(n+1\right)}{2\left(\sigma \left(n+1\right)+\sigma_{2}\left(n+1\right)\right)}.$$

In Equation (18), $\sigma_{2}\left(n+1\right)$ represents the standard deviation of the variations in the solar irradiance measurement vector *θ* between continuous time steps along a window of size *K*. Consequently, the vector of consecutive variations defined by $\Delta_{2}\left(n+1\right)$ is given by:

(19) $$\Delta_{2k}\left(n+1\right)=\theta \left(n+1-k\right)-\theta \left(n-k\right),\;k=1,\ldots ,k-1.$$

Thus, the corresponding mean and standard deviation are defined by:

(20) $${µ}_{2}\left(n+1\right)=\frac{1}{k-1}\sum_{k=1}^{k-1}\Delta_{2k}\left(n+1\right)$$

and

(21) $$\sigma_{2}\left(n+1\right)=\sqrt{\frac{1}{k-1}\sum_{k=1}^{k-1}{\left(\Delta_{2k}\left(n+1\right)-{µ}_{2}\left(n+1\right)\right)}^{2}}.$$

## 3. Proposed Multi-Source Adaptive Linear Energy Prediction Model (Adaptive LINE-P)

As described above, LINE-P is designed and developed based on sampling theory and approximation; we propose a novel adaptive linear energy prediction model (named Adaptive LINE-P), of which the main purpose is to add adaptive weighting to LINE-P. Rather than using a fixed weighting parameter, which makes it difficult to reflect the different properties of energy harvesters such as solar-based ones, Adaptive LINE-P is based on energy profiles, which improves the accuracy, adaptability, and reliability of the energy predictions.

We first present the Adaptive LINE-P model and evaluate its basic performance. Thereafter, we compare its performance against that of Pro-Energy, Pro-Energy-VLT, IPro-Energy, and UD-WCMA. We used four datasets of traces of harvested energy. These datasets are from trusted sources, and taken from different locations of the USA and Europe. Furthermore, three datasets for solar energy, i.e., Southern California Edison Company (SCE, Rosemead, CA, USA), Pacific Gas and Electric Company (PG&E, San Francisco, CA, USA), and San Diego Gas & Electric Company (SDG&E, Santiago, CA, USA) [16] are used; we also selected one dataset for wind energy from Elia (Belgium-based power generation company, Brussels, Belgium) [17].

### 3.1. Sampling Operators

Let us suppose that a function f is defined for every point of some domain D and has series representation there in the form:

$$f\left(t\right):=\overset{\infty }{\underset{k=-\infty }{\sum }}f\left(t_{k}\right)s_{k}\left(t\right),$$

in which {$t_{k}$} is a collection of points of D and {$s_{k}$} is some set of suitable expansion functions. Such an expansion is called a sampling series. The function f is represented in its entirety in terms of its values, that is samples, at a discrete subset of its domain. For the uniformly continuous and bounded f∈C(ℝ), the generalized sampling series are given by (t∈ℝ;w>0) as per Equation (22),

(22) $$\left(S_{w}f\right)\left(t\right):=\overset{\infty }{\underset{k=-\infty }{\sum }}f\left(\frac{k}{w}\right)s\left(wt-k\right),$$

where s∈C(ℝ) is a kernel function.

If the kernel function used in the sampling series is the cardinal sine, defined in the form:

$$s\left(t\right)=\sin c\left(t\right):=\frac{\sin\pi t}{\pi t},$$

we get the classical (Whittaker-Kotel’nikov-) Shannon sampling operator,

(23) $$\left(S_{w}^{\sin c}f\right)\left(t\right):=\overset{\infty }{\underset{k=-\infty }{\sum }}f\left(\frac{k}{w}\right)\sin c\left(wt-k\right).$$

Let us take w=1 and t=j∈ℤ in Equation (22), then

(24) $$\left(S_{1}f\right)\left(j\right):=\overset{\infty }{\underset{k=-\infty }{\sum }}f\left(k\right)s\left(j-k\right).$$

### 3.2. Kernels 

The general kernel for the sampling operators Equation (22) is expressed below.

**Definition** **1.** ([18]) *If* s:ℝ→ℂ *is a bounded function such that the absolute moment*

(25) $$m_{0}\left(s\right):=\overset{\infty }{\underset{k=-\infty }{\sum }}\left|s\left(u-k\right)\right|<\infty \left(u\in \mathbb{R}\right),$$

*with the absolute or positive convergence uniform on compact subsets of* ℝ*, and we have a partition of unity*

(26) $$\overset{\infty }{\underset{k=-\infty }{\sum }}s\left(u-k\right)=1\;\left(u\in \mathbb{R}\right),$$

*then* s *is called a kernel for sampling operators Equation (22)*.

The conditions in Definition 1 guarantee that the series in Equation (22) converges for f∈C(ℝ); moreover, we have uniform convergence

$$\left\|(S_{w}f\right)-f\|\to 0,\left(w\to \infty \right)$$

for any f∈C(ℝ). We estimate the speed of the convergence of sampling operators in terms of the modulus of smoothness $\omega_{r}$ (r∈ℕ) (see [18,19,20,21,22]) in the form

$$\|\left(S_{w}f\right)-f\|=M\omega_{r}\left(f,\frac{1}{w}\right),$$

where *M* is a positive constant. If we have an estimate in terms of a high order of modulus of smoothness, then the sampling operator rapidly converges for smooth signals.

The main aim of this article is to use for signal prediction the generalized sampling operators Equation (22), where the kernel function s is defined through the Fourier transform of a certain window function:

**Definition** **2.** *A function* λ∈C(ℝ) *is called a window function for a kernel of a sampling operator if* λ(0)=1 *and* λ(±2k)=0 *for* k∈ℕ.

Our kernel function is defined by the equality

(27) $$s\left(t\right):=s\;\left(\lambda ;t\right):=\frac{1}{2}\int_{-\infty }^{\infty }\lambda \left(u\right)\;\exp\left(-i\pi tu\right)du.$$

A special case of the kernel function are M-bandlimited kernels, defined by Equation (27), using window functions λ(u)=0 (|u|≥M>0). We consider the case *M* = 1, i.e., with kernels defined using window functions $\lambda \in C_{\left[-1,1\right]},\lambda \left(0\right)=1,\;\lambda \left(u\right)=0\;\left(\left|u\right|\geq 1\right)$. If the window function is an even function, then we get an even kernel:

(28) $$s\left(t\right)=\int_{0}^{1}\;\lambda \left(u\right)\cos\left(\pi tu\right)du.$$

Generally, for some cases, non-symmetric kernels are more suitable. In such cases, we prefer the general window function $\lambda \in C_{\left[-1,1\right]}$ and define the kernel in the form

(29) $$s\left(t\right)=\frac{1}{2}\int_{-1}^{1}\;\lambda \left(u\right)\;\exp\left(-i\pi tu\right)du.$$

These sorts of kernels arise in conjunction with window functions widely use in applications (e.g., [23,24,25,26]), particularly in signal analysis. Many kernels can be defined by Equation (28), e.g.,

(1) λ(u)=1 represents the sine function;
(2) $\lambda_{j}\left(u\right):=\cos\;\pi \left(j+\frac{1}{2}\right)u,\;j=0,1,2,\ldots$ defines the Rogosinski-type kernel (see [20]), in the form
  (30) $$r_{j}\left(t\right):=\frac{1}{2}\left(\sin c\left(t+j+\frac{1}{2}\right)+\sin c\left(t-j-\frac{1}{2}\right)\right)$$
  (31) $$=\frac{\frac{{\left(-1\right)}^{j}}{\pi }\left(j+\frac{1}{2}\right)}{{\left(j+\frac{1}{2}\right)}^{2}-t^{2}}\cos\pi t.$$
  Powers of the Hann window (see [25], Equation (25));
  (32) $$\lambda_{H,m}\left(u\right):=\cos^{m}\left(\frac{\pi u}{2}\right)$$
  give a general Hann kernel in the form
  (33) $$s_{H,m}\left(t\right)=2^{-m}\frac{\Gamma \left(1+m\right)}{\Gamma \left(1+\frac{m}{2}-t\right)\Gamma \left(1+\frac{m}{2}+t\right)},$$
  where Γ is the Euler gamma function.
  If m=1, we get the Hann kernel:
  (34) $$s_{H}\left(t\right)=\frac{\left(\sin c\left(t-1\right)+2\sin c\left(t\right)+\sin c\left(t+1\right)\right)}{4}.$$
(3) The general cosine window
  (35) $$\lambda_{C,a}\left(u\right):=\overset{n}{\underset{k=0}{\sum }}a_{k}\cos k\pi u,$$
  illustrates the Blackman–Harris kernel (see [20]),
  (36) $$s_{C,a}\left(t\right):=1/2\sum_{k=0}^{n}a_{k}\left(\sin c\left(t-k\right)+\sin c\left(t+k\right)\right),$$
  provided (here and following ⌊x⌋ is the largest integer less than or equal to x∈ℝ):
  (37) $$\overset{\left\lfloor \frac{n}{2}\right\rfloor }{\underset{k=0}{\sum }}a_{2k}=\overset{\left\lfloor \frac{n+1}{2}\right\rfloor }{\underset{k=0}{\sum }}a_{2k-1}=\frac{1}{2}.$$

We get the Hann window if we take n=1 in Equation (35), and the Blackman window if n=2 and $a_{0}=a$ in Equation (35). For n∈ℕ there exists a choice of parameters that allows us to have the order of approximation of the corresponding sampling operators estimated by high (2*n*) order of modulus of smoothness $\omega_{2n}\left(f;\frac{1}{w}\right)x$ (cf. [20]). Another possibility for the parameter vector $a=a^{*}$ in (35), where the parameter vector $a^{*}=\left(a_{0}^{*},a_{1}^{*},\ldots ,a_{n}^{*}\right)\in {\mathbb{R}}^{n+1}$ has components $a_{0}^{*}=\frac{1}{2^{2n}}\;\left(\begin{array}{c} 2n\\ n \end{array}\right)$ and $a_{k}^{*}=\frac{1}{2^{2n-1}}\;\left(\begin{array}{c} 2n\\ n-k \end{array}\right)$ for k=1,2,…,n, gives us by Equation (28) a family of rapidly decreasing kernels $s_{H,2n}=O(|t{|}^{2n+1})$ (see [21] for corresponding operator norms and [22] for truncation errors).

The general cosine window generates a linear combination of translated sine-functions; rather than the general cosine window, a window in the form Equation (38) can be used:

(38) $$\lambda_{E,a}\left(u\right):=\overset{n}{\underset{k=-n}{\sum }}a_{k}e^{ik\pi u}\;(n\in \mathbb{N},\;a=\left(a_{-n},a_{-n+1},\ldots ,\;a_{n})\right)\in {\mathbb{R}}^{2n+1},$$

provided

(39) $$\overset{\left\lfloor \frac{n}{2}\right\rfloor }{\underset{k=0}{\sum }}a_{2k}=\overset{\left\lfloor \frac{n+1}{2}\right\rfloor }{\underset{k=1}{\sum }}a_{2k-1}=\frac{1}{2}.$$

If we use Equation (29), we get a corresponding kernel in the form of Equation (40):

(40) $$s_{E,a}\left(t\right)=\overset{n}{\underset{k=-n}{\sum }}a_{k}\sin c\left(t-k\right),$$

which fulfills the properties of a kernel in terms of Definition 1, because the condition in Equation (39) guarantees that we have Equation (26) and that $m_{0}(s_{E,a})$ is bounded. Let us take w=1 and t=j∈ ℤ in Equation (22), then for kernel $s_{E,a}$ we get

(41) $$(S_{1;E,a}f)\left(j\right):=\overset{\infty }{\underset{k=-\infty }{\sum }}f\left(k\right)s_{E,a}\left(j-k\right)=\overset{n}{\underset{k=-n}{\sum }}a_{k}f\left(t-k\right).$$

#### Comment on Approximation Error Estimates

The reader can refer to this section in [5] for a short discussion of approximation error estimates. Note, for the case at hand: if we have for some *r* (r∈ℕ), an estimate of speed of convergence in terms of modulus of smoothness $\;\omega_{r},$ then the sampling series representation is exact for polynomials with a maximal power less than or equal to (*r* + 1).

### 3.3. Adaptive Predictors

We need adaptive predictors because the energy profiles can have different properties, i.e., with different smoothness, variation, etc. For different types of profiles we need different kernels for the sampling operators. In the current approach, we use the following kernels:

- For smooth profiles, kernels allow approximation order, estimates through high order of modulus of smoothness.
- For unstable profiles, the kernel provides a sampling operator with minimal (close to 1) norm.

Note: The trivial error estimate signal for additive noise is in the form $\left||S_{w}|\right|$·||v||, where $\left||S_{w}|\right|$ is the operator norm and ||v || is the norm of the noise component, i.e., if the operator norm is equal to 4, then in the worst case, we have 4-fold amplification of the noise in the predicted energy profile.

We deal with other profiles with a kernel that provides a sampling operator with good approximation properties and a small norm.

In order to choose the predictor kernel, we use $l_{1}$ norms of the prediction errors of previous estimates.

### 3.4. $l_{1}$ Norm

Now, we propose a method for adaptive prediction. We use the $l_{1}$ norms of the prediction errors. Moverover, we choose some *r* (r∈ℕ) kernels $s_{i}$ (*i* = 1, 2, …, *r*) that generate sampling operators with different properties (approximation order, norm, etc.) and compute the predicted values using it.

For predicting the *k*-th element, we choose the kernel for which the $l_{1}$ norm of the prediction errors for some one-sided neighborhood of the *k*-th element of the profile is minimal. We compute for the *k*-th element norms $||E_{i}\left(k\right)||1$ of errors in the following form:

$$||E_{i}\left(k\right)||1=\overset{n}{\underset{j=1}{\sum }}\left|f\left(k-j\right)-f_{p,i}\left(k-j\right)\right|,$$

where *f*(*k*) is the measured energy in slot *k* and $f_{p,i}\left(k\right)$ is the predicted energy for slot *k* using the kernels $s_{i}$.

For particular realization of the adaptive predictor, to cover different types of profiles, we choose three kernels with different approximation properties. We have corresponding sampling operators, a first one with minimal norm, a second one with a high order of approximation, and a third one with good approximation properties and a small norm.

### 3.5. Compressed Profiles

In this section, we suggest a method for compressing profile data to address the memory size limitation of WSN nodes. This is expressed as

$$\bar{f}\left(t\right)=\Sigma_{k}f\left(k\right)\bar{S\;}\left(t-k\right),$$

where $\bar{f}$ is the compressed profile, *f* the original profile, and $\bar{S}\left(t\right):=\frac{2}{\alpha }s\;\left(2t/\alpha \right)$ (α>0) the dilated kernel. Instead of *f*(*k*) we store $\bar{f}\left(\alpha k\right)$. For example if α=4, we use one-quarter of the memory.

For reconstruction, we use an interpolating kernel $\bar{S},$ i.e., a kernel defined using a window function that satisfies the equality

λ(u)+λ(1−u)=1, (u∈[0,1]).

The reconstruction formula is as follows:

$$f\left(j\right)\approx \Sigma_{k}\bar{f}\left(\alpha k\right)2\bar{S}\;\left(\frac{2}{\alpha }j-2k\right).$$

For efficient realizations we need to choose a reconstruction kernel, which allows us to compute a good enough reconstruction with a minimal number of operations; for the compression part the kernel may be more complicated, because we need to compress the profile only once a day. 

For a particular realization of the compression algorithm, we take α=4 and for both the kernel s and $\bar{S}$, we choose the Hann kernel (Equation (34)), which adds for reconstruction only three multiplications for every day in one prediction step. 

In what follows, we check the accuracy of Adaptive LINE-P (all cases). Thereafter, we can determine which of the cases is predicted best in terms of numerical value and suitable for further comparison with the state of the art.

### 3.6. Accuracy Evaluation of the Adaptive LINE-P (All Cases) Based on the MAE and MSE 

In this section we seek to find which of the three cases of Adaptive LINE-P model provides the most accuracy, robustness, lower errors, and adaptability in case of frequent changes in the energy source. In order to quantify the error in each case of Adaptive LINE-P, we consider two source energy profiles (solar and wind) and conduct various evaluations by means of MAE and MSE measures.

We have conducted two tests based on the two sources, namely, solar (SDG&E energy profile) and wind (Elia energy profile). Table 2 and Table 3 show the MAE and MSE for Adaptive LINE-P (all cases) for SDG&E energy profile for six individual days, as well as the average. Similarly, Table 4 and Table 5 show the MAE and MSE for Adaptive LINE-P (all cases) for Elia energy profile. 

Table 2, Table 3, Table 4 and Table 5 illustrate that, among all cases of Adaptive LINE-P model, Case III yields more accurate estimates as compared to Case I and Case II (error down by between −1% and −44%). Given this, Adaptive LINE-P model (Case III) has been selected for further comparison with the state of the art, as presented in the next section.

## 4. Comparative Analysis of Adaptive LINE-P (Case III, Non-Compressed Profiles) with the State of the Art

In this section, we assess the performance of Adaptive LINE-P (Case III) against that of UD-WCMA (the only other adaptive energy prediction model available in the literature) and against that of LINE-P (Case III) and IPro-energy (deemed the best two non-adaptive energy prediction models). Note that although we refer to LINE-P (Case III) and IPro-energy as non-adaptive, they are able to model energy variations, but they do not include specific adaptation mechanisms as in Adaptive LINE-P and UD-WCMA. 

The comparison is based on short, medium, and longer time period horizons. The classification of the time periods and their graphical representations are extracted from the real datasets available in [16,17]. For the longer time period, we consider 30 time slots; for the medium and shorter time periods we used 61 and 96 time slots in 42-, 22-, and 15-min data intervals in 24 h, respectively.

As far as the longer time period is concerned, by deploying real implementation of energy prediction, we have found that a longer time period such as a 1-h data interval is not sufficiently adaptive and feasible, specifically for those regions where the weather changes frequently. Therefore, we have reduced the data interval time from 60 to 42 min and conducted experiments based on 42-, 22-, and 15-min time periods.

In addition, we present the evaluation of the model based on the same two error measures as in Section 2, i.e., MSA and MSE; thereafter, we assess their time complexities and finally the analysis of the proposed compression technique.

### 4.1. Graphical Representation of Adaptive LINE-P (Case III) as Compared to the State of the Art Based on Longer, Medium, and Shorter Time Period Horizons

For the assessment of all the prediction models, we present the estimation errors in Table 6 and Table 7 by using MAE and MSE with the same profile PG&E available in [16]. 

Table 6 and Table 7 illustrate that adaptive LINE-P (Case III) yield less error comparatively to the other prediction models, except LINE-P (Case III), although the error difference between adaptive LINE-P and non-adaptive LINE-P is negligible. Actually, the estimation error is also dependent on the profile; in another profile, we have found higher error in non-adaptive LINE-P (Case III) than adaptive LINE-P.

**Figure 2:**
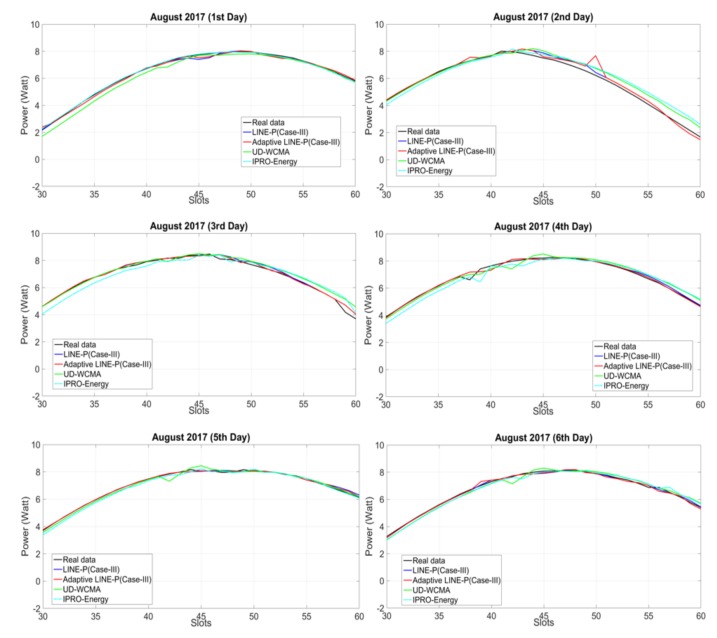
Graphical representation of Adaptive LINE-P (Case III) and state of the art based on the longer (42-min data interval) time period horizon for the solar profile with 30 time slots in 24 h.

To obtain the results shown in Figure 2, we have used a PG&E profile [16] for the solar energy to assess the adaptive LINE-P (Case III) and the state-of-the-art models based on longer (30 time slots) time period horizon. Figure 2 shows that the profile corresponds to highly consistent weather; all together, all days are nearly identical. 

However, some of the EP models are unable to predict the energy with full accuracy. For instance, UD-WCMA overestimates the energy on all six days. Even though for the first day it starts by underestimating, after the 40th time slot it estimated the real data well; after the 50th time slot it starts overestimating again. Therefore, this overestimation may indicate that UD-WCMA is not sufficiently adaptable, even for consistent profiles. 

Similarly, IPRO-Energy starts by underestimating at all days (except for the first day), but as compared to UD-WCMA, IPRO-Energy is better at modeling energy variations. Although IPRO-Energy and LINE-P (Case III) provide adequate results, both are based on a fixed weighting parameter factor; thus, they are not well suited for various types of solar energy harvesters, as mentioned earlier in the paper. On the other hand, Adaptive LINE-P (Case III) is not dependent on any fixed weighting parameter; it performs predictions on the adaptive weighting factor based on the profiles. Furthermore, we observe in Figure 2 (for all days) that the adaptive LINE-P (Case III) is highly accurate. Thus, for this energy profile, Adaptive LINE-P provides both accuracy and adaptability. 

In the following, we evaluate the performance of Adaptive LINE (Case III) along with the state of the art based on the medium (61 time slots) time period horizon of the solar energy profile SCE [16].

For further assessment of all the prediction models, we present the estimation errors in Table 8 and Table 9 by using MAE and MSE with the same profile SCE available in [16].

In relation to Table 8 and Table 9, it is shown that adaptive LINE-P (Case III) has less error possibility as compared to the other prediction models.

**Figure 3:**
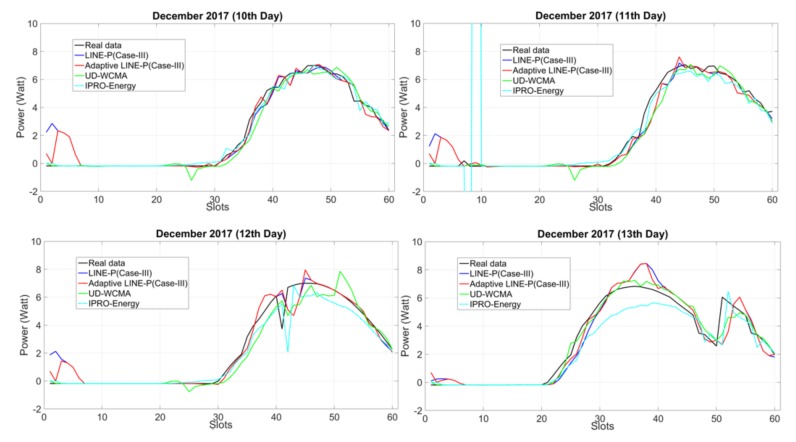
Graphical representation of Adaptive LINE-P (Case III) and state of the art based on the medium (22-min data interval) time period horizon of the solar profile with 61 time slots in 24 h.

Figure 3 shows the results for fairly consistent profiles, to see the behavior and adaptability of the prediction models based on the medium (61 time slots) time period horizon. The graphical representation shows that most of the models are estimating up to the mark only in the first day. It can be seen that in all other days, LINE-P (Case III) starts overestimating. UD-WCMA also starts overestimating in all days, especially on the 12th and 13th of December from the 45th to the 60th time slots, and 20th to 50th time slots. UD-WCMA yields the worst results comparative to the other prediction models. Furthermore, on 11th December, the IPro-Energy model is off the chart from the fifth to the 10th time slots. Although gradually its estimation is approaching the real data, it then starts underestimating from the 40th until the 53rd time slots. On the contrary, Adaptive LINE-P (Case III) seems much better and most of the time yields estimates close to the real data. 

In addition, we have assessed Adaptive LINE-P (Case III) comparative to the state of the art based on the graphical representation and classical error-calculating methods (MSA and MSE), as presented in what follows.

**Figure 4:**
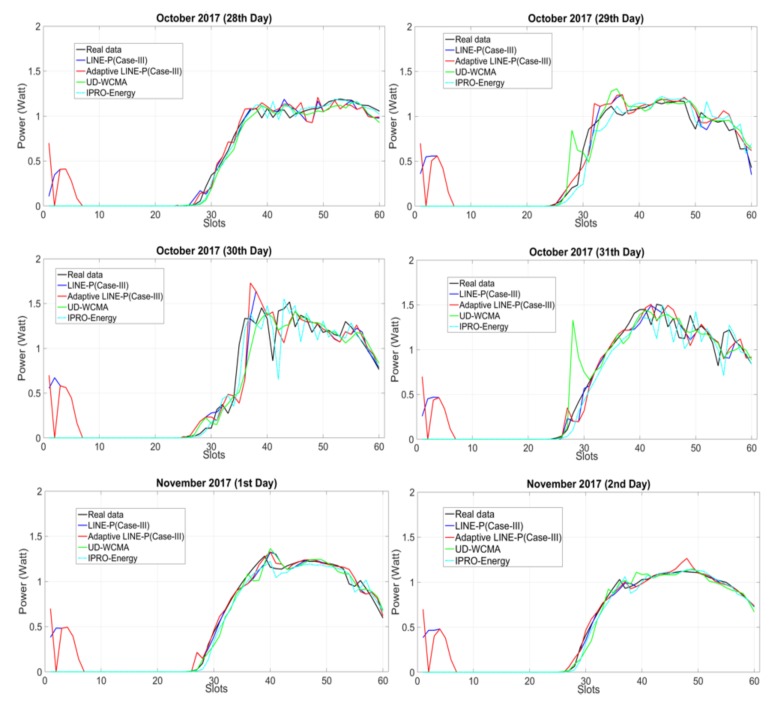
Graphical representation of Adaptive LINE-P (Case III) and the state of the art based on the medium (22-min data interval) time period horizon for the solar energy profile with 61 time slots in 24 h.

Figure 4 shows the comparison of Adaptive LINE-P (Case III) and the state of the art for the SDG&E solar energy profile. This profile exhibits very low power production throughout the figure. In addition, this profile is for cloudy days with lots of variation. This kind of profile is a real challenge for the prediction models; indeed, too much fluctuation and extremely sharp variation-based weather is difficult to predict accurately and requires continuous adaptation. As can be observed, most of the models, especially from the second to the fourth days, are overestimating. Moreover, UD-WCMA shows poor prediction for all the days. Furthermore, due to rapid changes in the profile, IPRO-Energy underestimates on the 30th and 31st of October, but once the profile becomes smooth IPRO-Energy predicts well until the end of profile, especially in the last two days. LINE-P (Case III) and Adaptive LINE-P (Case III) overestimate from the 30th to 40th time slots in all days. Thereafter, it can be observed that Adaptive LINE-P (Case III) also shows robustness and predicts accurately as compared to LINE-P (Case III) and the state of the art. 

For further assessment of all the prediction models, we also present the estimation errors in Table 10 and Table 11 by using MAE and MSE with the same profile SDG&E available in [16].

For this kind of energy profile, we found that in terms of MAE, Adaptive LINE-P (Case III) yields less errors than IPRO Energy and UD WCMA (between ca. −5% and ca. −34%), and is almost identical to LINE-P (Case III) (+0.5%). In terms of MSE, Adaptive LINE-P (Case III) yields lower error as compared to all other energy prediction models (between −5.5% and ca. −59%). In addition, Adaptive LINE-P (Case III) model is highly adaptive, as can be observed in Figure 4. However, LINE-P and IPRO-Energy also show good estimations, but they still suffer from the fixed weighting parameter issue, which was discussed earlier.

#### Results for Wind Energy

In what follows, we compare Adaptive LINE (Case III) with the state of the art based on the shorter (96 time slots in 24 h) time period horizon for the wind energy profile Elia available in [11].

**Figure 5:**
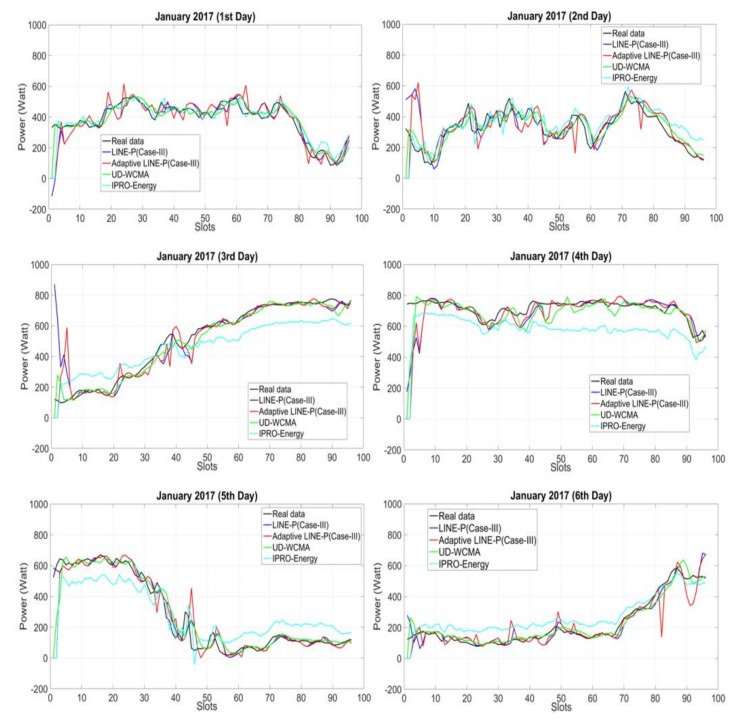
Graphical representation of Adaptive LINE-P (Case III) and state of the art based on the shorter (15-min data interval) time period horizon for the wind energy profile with 96 time slots in 24 h.

As shown in Figure 5, it can be observed that LINE-P (Case III) initially overestimates and then approaches the real data. Similar to the solar energy case, UD-WCMA predictions are rather far from the real data. Most of the time, it can be seen that it starts with an overestimation if the real data increases; on the other hand, if the real data decreases, then its behavior changes completely and underestimates, especially in the last two days in Figure 4. IPRO-Energy is also not yielding very good estimates. It is clearly visible in Figure 4 that both UD-WCMA and IPRO-Energy are not suitable for uncontrollable energy sources on the shorter time period horizon. On the other hand, Adaptive LINE-P (Case III) shows robustness, adaptability, suitability for variable-length time slots, and accuracy. If the profiles are changing frequently, then UD-WCMA and IPRO-Energy are not as accurate as Adaptive LINE-P (Case III). In addition, the Adaptive LINE-P (Case III) model is highly adaptable, as can be observed in Figure 5.

In the following section, we compute the accuracy of the adaptive LINE-P (Case III) by means of the MAE and MSE for the wind energy profile.

### 4.2. Accuracy Assessment of the Energy Prediction Models Based on the MAE and MSE for Solar and Wind Energy Profiles

In this section, we examine the models based on the multiple (solar and wind) energy profiles. In order to calculate the error possibility in Adaptive LINE-P (Case III) and the other energy prediction models, we consider the PG&E solar energy profile available in [16].

Table 12 and Table 13 illustrate that the results provided by Adaptive LINE-P (Case III) have less errors as compared to the other prediction models (down by up to −82%), with the exception of LINE-P (Case III) (+50%) in terms of MSE (Table 13).

Next, we deal with Elia wind energy profile [17] to see the possible errors in Adaptive LINE-P (Case III) as compared to the state of the art.

Similarly, Table 14 and Table 15 show that the proposed Adaptive LINE-P (CASE III) performs better than the other energy prediction models (error down by up to ca. −78% in terms of MAE and MSE). In the above section, we have compared the energy prediction models with two different sources, namely solar and wind data profiles; apart from a minor exception, the results show that Adaptive LINE-P (Case III) provides the best results as compared to the other energy prediction models. 

In the next section, we evaluate the performance of the proposed compressed profile method as compared to the state of the art. 

## 5. Comparison of the Compressed Profile Method with the State of the Art Based on the Shorter Time Period Horizon 

**Figure 6:**
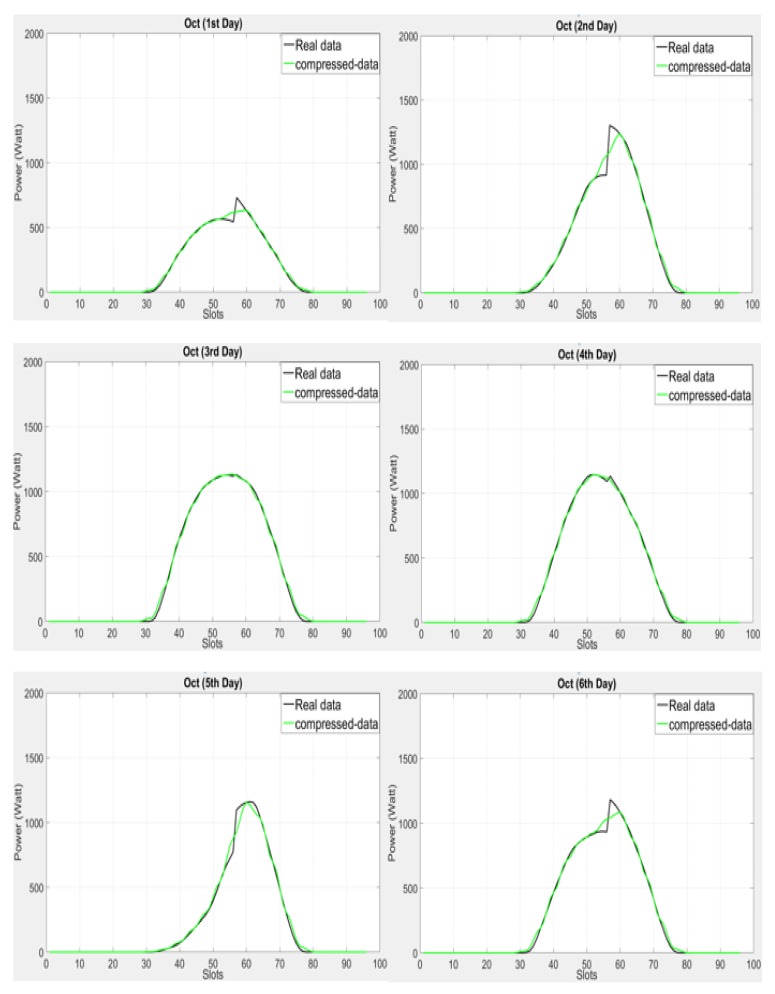
Graphical representation of compressed profile method against the real data based on the shorter (15-min data interval) time period horizon of solar energy profile (Elia) with 96 time slots in 24 h.

**Figure 7:**
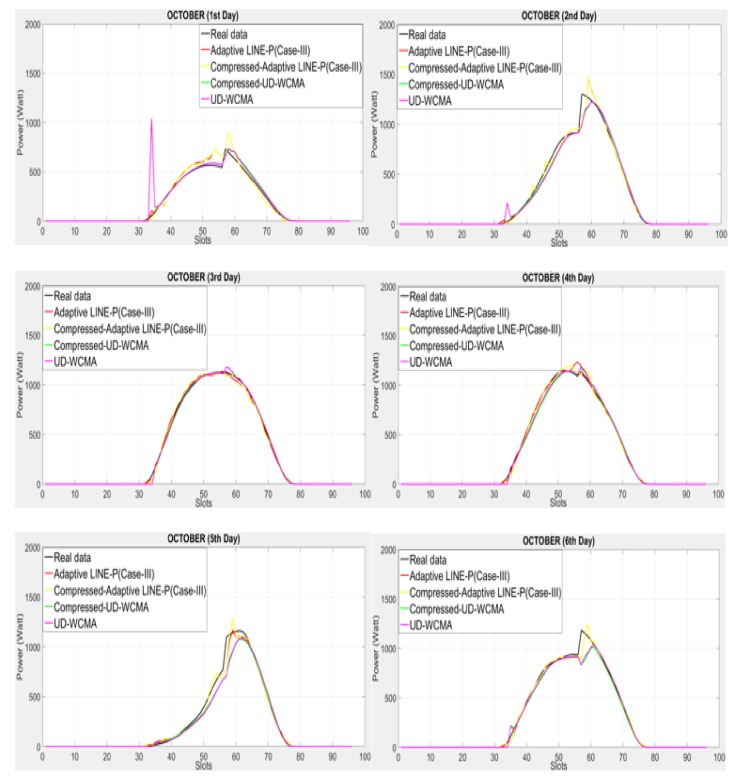
Graphical representation of the compressed profile method against the real data based on the shorter (15-min data interval) time period horizon of solar profile with 96 time slots in 24 h.

Here, we assess the compressed profile method in two steps. Firstly, in order to verify its accuracy and adaptability, we compare it with the real data (real profile), see Figure 6 and Figure 7. Secondly, we incorporate the method with the two adaptive energy prediction models (Adaptive LINE-P and UD-WCMA) for further assessment against their non-compressed versions, as well as against the real data. 

In all experiments, we use the shorter (96 time slots) time period horizon in 24 h. We check the accuracy of the compressed profile method against the graphical representation and a MAE and MSE as well.

### 5.1. Graphical Representation of the Compressed Profile Method and Its Error Estimation as Compared to the Real Data (a Real Dataset) Based on the Solar Energy Profile

In Figure 6, the energy profile reflects consistent weather; however, there are certain variations in each day. In this figure, the accuracy remains at a high level, though some lack in adaptability against the sharp variation is visible on all days (except on the third day). 

For further analysis of the proposed compressed profile method, we incorporate it into UD-WCMA and adaptive LINE-P (Case III) models to validate its accuracy when used with those models. In addition, we have used the same energy profile [17] to compare the error estimation with the non-compressed UD-WCMA and adaptive LINE-P (Case III) as well. The results for the solar energy profile are presented in Section 5.2 and those for the wind energy profile in Section 5.3.

### 5.2. Error Estimation with and without Compression Method in UD-WCMA and Adaptive LINE-P (Case III) for the Solar Energy Profile

Furthermore, Table 16 and Table 17 illustrate the error estimation with and without the proposed profile compression method in terms of MAE and MSE for the solar energy profile. 

It can be observed that incorporating the compressed profile method increases the MAE for UD-WCMA and Adaptive LINE-P (CASE III) by ca. +3.14% and +4.7%, respectively. Interestingly, it can also be seen that incorporating the compressed profile method increases the MSE by ca. +1.4% for UD-WCMA and decreases it by ca. −26% for Adaptive LINE-P. As seen earlier in the paper, the compressed profile method reduces the memory requirements by a factor of 2, thus offering a good trade-off between accuracy and memory requirements. In the following, we further evaluate the compressed profile method with the graphical representation as well as with MAE and MSE based on the wind energy profile.

### 5.3. Error Estimation with and without Compression Method in UD-WCMA and Adaptive LINE-P (Case III) for the Wind Energy Profile

Here, we use the wind energy profile to evaluate the performance of the compressed profile method as compared to the real data in terms of the graphical view. 

**Figure 8:**
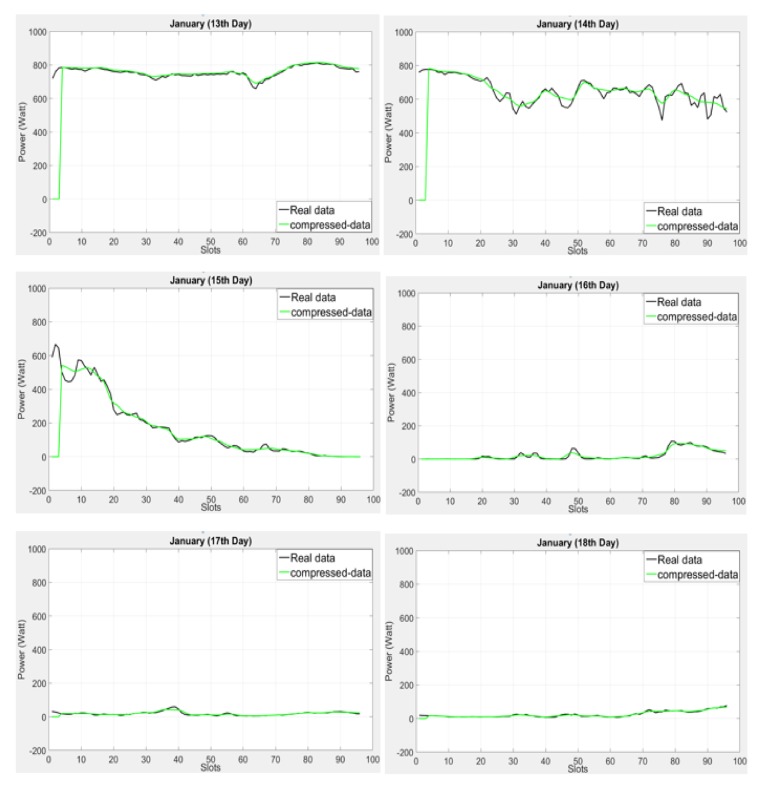
Graphical representation of the compressed profile method against the real data based on the shorter (15-min data interval) time period horizon of wind profile (Elia) with 96 time slots in 24 h.

Figure 8 exhibits extremely inconsistent weather; moreover, the last three days show low power productivity. As can been observed in the figure, the compressed profile method shows stability (smoothness) rather than adaptability, especially on 14th and 15th January, due to sharp variation; however, approaching the profile’s end, we see the robustness of the compressed profile method.

In the following, we present the prediction error of the compressed profile method in terms of MAE and MSE for the wind energy profile. 

### 5.4. Graphical Representation of the Prediction Model with and without the Compressed Profile Method for the Wind Energy Profile

In the following, we calculate the error estimation in terms of MAE and MSE for the wind energy profile.

**Figure 9:**
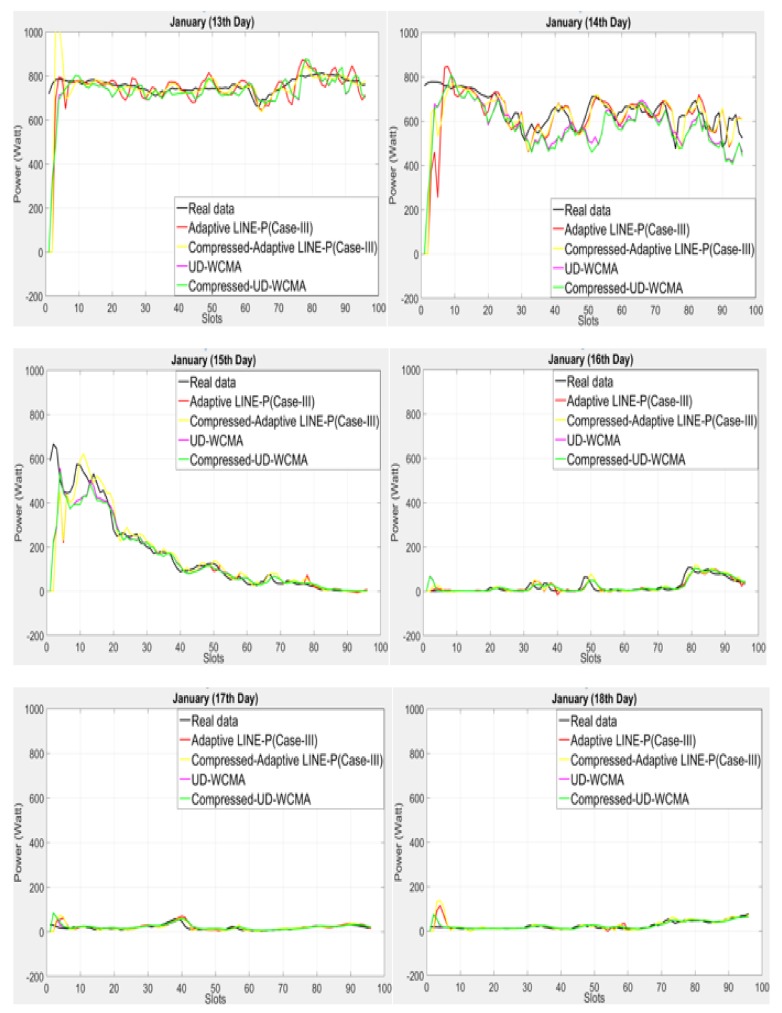
Graphical representation of energy prediction models with and without the compressed profile method based on the short time period horizon of the wind energy profile (Elia).

In Figure 9 we observed that the proposed compressed profile method integrated with Adaptive LINE-P (Case-III) and UD-WCMA shows stability and accuracy similar to the non-compressed Adaptive LINE-P (Case-III) and UD-WCMA; however, we calculated minor error in the compressed method as compared to the non-compressed method.

As can be observed in Table 18 and Table 19, the MAE and MSE values are of the same order of magnitude as for the solar energy profile (Table 16 and Table 17). Next, for further evaluation, we apply the same profile we used above for the compressed profile method into the UD-WCMA and Adaptive Line-P (CASE III) energy prediction models and then compare their results without adding the compression feature.

In addition, Table 18 and Table 19 illustrate the error estimation with and without the proposed profile compression method in terms of MAE and MSE for the wind energy profile. In Table 18, it can be observed that incorporating the compressed profile method increases the MAE for UD-WCMA by ca. +0.52% but decreases it for Adaptive LINE-P (CASE III) by ca. −6.3%. 

In Table 19, it can be seen that incorporating the compressed profile method increases the MAE for UD-WCMA by +1.5% but decreases it for Adaptive LINE-P (CASE III) by ca. −7.93%. As seen earlier in the paper, the compressed profile method reduces the memory requirements by a factor of 2. In line with the results shown for the solar energy profile (Table 16 and Table 17), the results for the wind energy profile show that that the proposed compressed profile method offers a good trade-off between accuracy and memory requirements.

## 6. Conclusions

We have presented Adaptive LINE-P (three cases-based) prediction model for multi-source (solar and wind) energy sources. The proposed model is independent of the fixed length time slot and fixed weighting parameter. We have conducted experiments with three time period horizons (shorter, medium, and longer) with different time slots. Adaptive LINE-P model chooses the weighting parameter based on the actual energy profile. We have conducted numerous experiments with real datasets, and for the error evaluation we have used the MAE and MSE error calculating method. The results show that Adaptive LINE-P, especially (Case III), is 90–94% accurate (depending on the weather). In addition, our prediction model is highly adaptable against sharp variations as compared to other adaptive and non-adaptive prediction models. Most of the time, the proposed Adaptive LINE-P model yields estimates with less errors, except in a few cases, depending on the energy profile. Nevertheless, this is a small (and relatively rare) price to pay as compared to the general gains offered by the adaptive feature of the model. Moreover, we proposed a compressed profile method that can easily be incorporated into any prediction model; this method allows us to reduce the memory requirements by 50% and provides 90% accuracy.

In the future, we plan to work on the data prediction concept, which is also one of promising solution for saving energy. This concept can be applied to cases where the data are identical for longer time periods, for instance temperature and humidity, specifically in the domain of WSNs.

## Figures and Tables

**Table 1:** Prediction error in terms of MAE and MSE for IPro-Energy and Pro-Energy.

Energy Prediction Model	MAE (Mean Absolute Error)	MSE (Mean Square Error)
IPro-Energy	0.09915	0.0608
Pro-Energy	0.15875	0.1665

**Table 2:** Error comparison of adaptive LINE-P (all cases) based on the MAE for SDG&E solar energy profile.

MODELS	MAE (Day 1)	MAE (Day 2)	MAE (Day 3)	MAE (Day 4)	MAE (Day 5)	MAE (Day 6)	Average MAE
Adaptive LINE-P (Case I)	0.0632	0.1071	0.1575	0.0969	0.0597	0.0382	0.0871
Adaptive LINE-P (Case II)	0.0591	0.1148	0.1993	0.1239	0.0869	0.0554	0.1065
Adaptive LINE-P (Case III)	0.0634	0.0887	0.1486	0.0902	0.0523	0.033	0.0793

**Table 3:** Error comparison of adaptive LINE-P (all cases) based on the MSE for SDG&E solar energy profile.

MODELS	MSE (Day 1)	MSE (Day 2)	MSE (Day 3)	MSE (Day 4)	MSE (Day 5)	MSE (Day 6)	Average MSE
Adaptive LINE-P (Case I)	0.0212	0.0649	0.1462	0.0588	0.0184	0.0069	0.0527
Adaptive LINE-P (Case II)	0.0180	0.0698	0.2402	0.0769	0.0356	0.0207	0.0768
Adaptive LINE-P (Case III)	0.0161	0.0395	0.1287	0.0537	0.0133	0.0065	0.0429

**Table 4:** Error comparison of adaptive LINE-P (all cases) based on the MAE for Elia wind energy profile.

MODELS	MAE (Day 1)	MAE (Day 2)	MAE (Day 3)	MAE (Day 4)	MAE (Day 5)	MAE (Day 6)	Average MSE
Adaptive LINE-P (Case I)	0.0984	0.1455	0.0494	0.0391	0.1192	0.0806	0.0887
Adaptive LINE-P (Case II)	0.0902	0.1598	0.0343	0.0295	0.1481	0.0754	0.0895
Adaptive LINE-P (Case III)	0.0955	0.1444	0.0474	0.0322	0.1137	0.0921	0.0875

**Table 5:** Error comparison of adaptive LINE-P (all cases) based on the MSE for Elia wind energy profile.

MODELS	MSE (Day 1)	MSE (Day 2)	MSE (Day 3)	MSE (Day 4)	MSE (Day 5)	MSE (Day 6)	Average MSE
Adaptive LINE-P (Case I)	0.0185	0.0410	0.0070	0.0038	0.0445	0.0129	0.0212
Adaptive LINE-P (Case II)	0.0149	0.0484	0.0027	0.0019	0.0597	0.0106	0.0230
Adaptive LINE-P (Case III)	0.0156	0.0412	0.0031	0.0021	0.0431	0.0211	0.0210

**Table 6:** Error comparison of prediction models based on MAE for the PG&E solar energy profile.

MODELS	MAE (Day 1)	MAE (Day 2)	MAE (Day 3)	MAE (Day 4)	MAE (Day 5)	MAE (Day 6)	Average MAE
LINE-P (Case III)	0.0269	0.0315	0.0188	0.0189	0.0138	0.0141	0.0206
UD-WCMA	0.0407	0.0686	0.0685	0.0330	0.0194	0.0312	0.0435
IPRO-Energy	0.0070	0.0861	0.0645	0.0526	0.0252	0.0310	0.0443
Adaptive LINE-P (Case III)	0.0251	0.0370	0.0150	0.0210	0.0171	0.0155	0.0217

**Table 7:** Error comparison of prediction models based on the MSE by deploying PG&E solar profile.

MODELS	MSE (Day 1)	MSE (Day 2)	MSE (Day 3)	MSE (Day 4)	MSE (Day 5)	MSE (Day 6)	Average MSE
LINE-P (Case III)	0.0037	0.0040	0.0018	0.0017	0.0011	0.0008	0.0021
UD-WCMA	0.0063	0.0184	0.0429	0.0040	0.0017	0.0034	0.0127
IPRO-Energy	0.0002	0.0267	0.0127	0.0037	0.0027	0.0032	0.0082
Adaptive LINE-P (Case III)	0.003	0.0086	0.0013	0.0037	0.0015	0.0011	0.0032

**Table 8:** Error comparison of the prediction models based on MAE for the SCE solar energy profile.

MODELS	MAE (Day 1)	MAE (Day 2)	MAE (Day 3)	MAE (Day 4)	Average MAE
LINE-P (Case III)	0.0820	0.0945	0.0944	0.1563	0.1068
UD-WCMA	0.1088	0.1347	0.1881	0.0979	0.1323
IPRO-Energy	0.0782	0.0842	0.1745	0.2008	0.1344
Adaptive LINE-P (Case III)	0.0802	0.0970	0.0932	0.1405	0.1027

**Table 9:** Error comparison of the prediction models based on MSE for the SCE solar energy profile.

MODELS	MSE (Day 1)	MSE (Day 2)	MSE (Day 3)	MSE (Day 4)	Average MSE
LINE-P (Case III)	0.0348	0.0493	0.0850	0.1051	0.0685
UD-WCMA	0.0559	0.0797	0.1509	0.0465	0.0832
IPRO-Energy	0.0313	0.0351	0.1898	0.1524	0.1021
Adaptive LINE-P (Case III)	0.0352	0.0530	0.0849	0.0905	0.0659

**Table 10:** Error comparison of the prediction models based on MAE for the SDG&E solar energy profile.

MODELS	MAE (Day 1)	MAE (Day 2)	MAE (Day 3)	MAE (Day 4)	MAE (Day 5)	MAE (Day 6)	Average MAE
LINE-P (Case III)	0.0619	0.0841	0.1432	0.0910	0.0668	0.0362	0.0805
UD-WCMA	0.0625	0.1224	0.1376	0.1409	0.1392	0.1381	0.1234
IPRO-Energy	0.0467	0.1053	0.1305	0.1141	0.0733	0.0418	0.0852
Adaptive LINE-P (Case III)	0.0645	0.0861	0.1486	0.0976	0.0525	0.0366	0.0809

**Table 11:** Error comparison of the prediction models based on MSE for the SDG&E solar energy profile.

MODELS	MSE (Day 1)	MSE (Day 2)	MSE (Day 3)	MSE (Day 4)	MSE (Day 5)	MSE (Day 6)	Average MSE
LINE-P (Case III)	0.0156	0.0392	0.1378	0.0417	0.0193	0.0066	0.0433
UD-WCMA	0.0160	0.1040	0.1185	0.1220	0.1386	0.1281	0.1045
IPRO-Energy	0.0115	0.0555	0.1150	0.0615	0.0198	0.0091	0.0454
Adaptive LINE-P (Case III)	0.0161	0.0395	0.1287	0.0537	0.0133	0.0065	0.0429

**Table 12:** Error comparison of the energy prediction models in terms of MAE for the solar energy profiles.

MODELS	MAE (Day 1)	MAE (Day 2)	MAE (Day 3)	MAE (Day 4)	MAE (Day 5)	MAE (Day 6)	Average MAE
LINE-P (Case III)	0.0269	0.0315	0.0188	0.0189	0.0138	0.0141	0.0206
UD-WCMA	0.0407	0.0686	0.0685	0.0330	0.03476	0.03688	0.047
IPRO-Energy	0.0070	0.0861	0.0645	0.0526	0.0252	0.0310	0.044
Adaptive LINE-P (Case III)	0.0251	0.037	0.0150	0.0210	0.0171	0.0155	0.0217

**Table 13:** Error comparison of the energy prediction models in terms of MSE for the solar energy profile.

MODELS	MSE (Day 1)	MSE (Day 2)	MSE (Day 3)	MSE (Day 4)	MSE (Day 5)	MSE (Day 6)	Average MSE
LINE-P (Case III)	0.0037	0.0040	0.0018	0.0017	0.0011	0.0008	0.0021
UD-WCMA	0.0049	0.0184	0.0429	0.0040	0.0329	0.0372	0.0233
IPRO-Energy	0.0002	0.0267	0.0127	0.0037	0.0027	0.0032	0.0082
Adaptive LINE-P (Case III)	0.003	0.0086	0.0013	0.0095	0.0015	0.0011	0.0041

**Table 14:** Error comparison of the energy prediction models in terms of MAE for the wind energy profile.

MODELS	MAE (Day 1)	MAE (Day 2)	MAE (Day 3)	MAE (Day 4)	MAE (Day 5)	MAE (Day 6)	Average MAE
LINE-P (Case III)	0.0349	0.0623	0.1083	0.4257	0.2294	0.1565	0.1695
UD-WCMA	0.0330	0.0879	0.1088	0.3437	0.1946	0.1279	0.1493
IPRO-Energy	0.0986	0.1907	0.1863	0.6094	3.0968	0.4993	0.7801
Adaptive LINE-P (Case III)	0.0338	0.0569	0.1095	0.4186	0.2133	0.1594	0.16525

**Table 15:** Error comparison of the energy prediction models in terms of MAE for the wind energy profile.

MODELS	MSE (Day 1)	MSE (Day 2)	MSE (Day 3)	MSE (Day 4)	MSE (Day 5)	MSE (Day 6)	Average MSE
LINE-P (Case III)	0.0021	0.0065	0.0311	0.4667	0.1451	0.0545	0.1176
UD-WCMA	0.0018	0.0144	0.0415	0.3143	0.1048	0.0323	0.0845
IPRO-Energy	0.0112	0.0441	0.0936	0.5489	1.9788	0.3059	0.4970
Adaptive LINE-P (Case III)	0.0020	0.0061	0.0292	0.4243	0.1278	0.0563	0.1076

**Table 16:** Error estimation MAE of the prediction models with and without the compressed profile for the solar energy profile.

MODELS	MAE (Day 1)	MAE (Day 2)	MAE (Day 3)	MAE (Day 4)	MAE (Day 5)	MAE (Day 6)	Average MAE
Compress-Method	0.0471	0.0550	0.0248	0.0283	0.0608	0.0432	0.0432
Compressed-UD-WCMA	0.1225	0.0719	0.0265	0.0430	0.1046	0.0649	0.0722
Non-Compressed-UD-WCMA	0.1138	0.0697	0.0241	0.0407	0.1130	0.0588	0.0700
Compressed-Adaptive LINE-P (Case III)	0.1327	0.0687	0.0305	0.0360	0.0771	0.0431	0.0646
Non-Compressed-Adaptive LINE-P (Case III)	0.1315	0.0793	0.0264	0.0258	0.0677	0.0398	0.0617

**Table 17:** Error estimation MSE of the prediction models with and without the compressed profile for the solar energy profile.

MODELS	MSE (Day 1)	MSE (Day 2)	MSE (Day 3)	MSE (Day 4)	MSE (Day 5)	MSE (Day 6)	Average MSE
Compress-Method	0.0121	0.0177	0.0022	0.0024	0.0202	0.0087	0.0105
Compressed–UD-WCMA	0.3549	0.0298	0.0030	0.0077	0.0833	0.0351	0.0856
Non-Compressed-UD-WCMA	0.3528	0.0324	0.0025	0.0055	0.0818	0.0316	0.0844
Compressed-Adaptive LINE-P (Case III)	0.0691	0.0306	0.0033	0.0052	0.0342	0.0156	0.0263
Non-Compressed-Adaptive LINE-P (Case III)	0.1315	0.0302	0.0022	0.0046	0.0293	0.0152	0.0355

**Table 18:** Error estimation in terms of MAE of the prediction models with and without the compressed profile method for the wind energy profile.

MODELS	MAE (Day 1)	MAE (Day 2)	MAE (Day 3)	MAE (Day 4)	MAE (Day 5)	MAE (Day 6)	Average MAE
Compressed-profile	0.0089	0.0303	0.0577	0.2216	0.1189	0.0704	0.0846
Compressed-UD-WCMA	0.0761	0.1186	0.0431	0.0424	0.0997	0.0797	0.0766
Non-Compressed-UD-WCMA	0.0757	0.1179	0.0425	0.0405	0.1008	0.0799	0.0762
Compressed-Adaptive LINE-P (Case III)	0.0813	0.1322	0.0435	0.0280	0.1137	0.0921	0.0818
Non-Compressed-Adaptive LINE-P (Case III)	0.0955	0.1444	0.0474	0.0322	0.1120	0.0927	0.0873

**Table 19:** Error estimation in terms of MSE of the prediction models with and without the compressed profile method for the wind energy profile.

MODELS	MSE (Day 1)	MSE (Day 2)	MSE (Day 3)	MSE (Day 4)	MSE (Day 5)	MSE (Day 6)	Average MSE
Compressed-profile	0.0001	0.0019	0.0088	0.1146	0.0398	0.0107	0.0293
Compressed-UD-WCMA	0.0102	0.0233	0.0039	0.0038	0.0267	0.0132	0.0135
Non-Compressed-UD-WCMA	0.0101	0.0230	0.0038	0.0035	0.0267	0.0132	0.0133
Compressed-Adaptive LINE-P (Case III)	0.0145	0.0405	0.0065	0.0016	0.0432	0.0333	0.0232
Non-Compressed-Adaptive LINE-P (Case III)	0.0186	0.0456	0.0074	0.0021	0.0441	0.0336	0.0252
